# Thin Magnetically Soft Wires for Magnetic Microsensors

**DOI:** 10.3390/s91109216

**Published:** 2009-11-18

**Authors:** Valentina Zhukova, Mihail Ipatov, Arcady Zhukov

**Affiliations:** 1 Dpto. Física de Materiales, Facultad de Química, UPV/EHU, P.O. Paseo Manuel de Lardizabal, 3, 20018, San Sebastián, Spain; E-Mails: valentina.zhukova@ehu.es (V.Z.); mihail.ipatov@ehu.es (M.I.); 2 TAMAG Ibérica S.L., Parque Tecnológico de Miramón, Paseo Mikeletegi 56, 1ª Planta, 20009 San Sebastián, Spain

**Keywords:** glass coated microwires, Barkhausen jump, magnetization curves

## Abstract

Recent advances in technology involving magnetic materials require development of novel advanced magnetic materials with improved magnetic and magneto-transport properties and with reduced dimensionality. Therefore magnetic materials with outstanding magnetic characteristics and reduced dimensionality have recently gained much attention. Among these magnetic materials a family of thin wires with reduced geometrical dimensions (of order of 1–30 μm in diameter) have gained importance within the last few years. These thin wires combine excellent soft magnetic properties (with coercivities up to 4 A/m) with attractive magneto-transport properties (Giant Magneto-impedance effect, GMI, Giant Magneto-resistance effect, GMR) and an unusual re-magnetization process in positive magnetostriction compositions exhibiting quite fast domain wall propagation. In this paper we overview the magnetic and magneto-transport properties of these microwires that make them suitable for microsensor applications.

## 1. Introduction

Recent tendencies towards miniaturization of modern magnetic sensors and devices has stimulated development of functional magnetic materials with reduced dimensionality and improved magnetic and magneto-transport properties. Certain progress has been recently achieved in the fabrication of novel magnetic nano-materials (thin films, nanowires, nano-dots…); although quite sophisticated technology must be used, on many occasions the magnetic properties of these materials are rather poorer than the properties of bulk magnetic materials (amorphous ribbons, wires, sintered materials…) and the fabrication process is much more expensive and complex [1,2]. On the other hand, industrial sectors such as magnetic sensors, microelectronics, security, *etc.*, need cheap materials with reduced dimensionality but which still possess high magnetic properties (particularly enhanced magnetic softness). Therefore magnetic materials with outstanding magnetic characteristics and reduced dimensionality have recently gained much attention.

Among these modern magnetic materials a family of thin wires with reduced geometrical dimensions (on the order of 1–30 μm in diameter) have gained importance within the last few years [1-4]. These thin wires combine excellent soft magnetic properties (with coercivities up to 4 A/m) with attractive magneto-transport properties (Giant Magneto-impedance effect, GMI, Giant Magneto-resistance effect, GMR) and an unusual re-magnetization process in positive magnetostriction compositions exhibiting magnetically bistable behaviour and quite fast domain wall propagation [2-6]. Among the various scientific groups involved in development of thin magnetic wires and studies of their magnetic properties suitable for micro-sensors, Spanish groups play a particularly important role [2-5] in research on this topic.

It is now nearly 50 years since the first metallic glass (amorphous metal) was produced by rapid quenching from the liquid state by I.S. Miroshnitchenko and I.V. Salli [7] and later by P. Duwez *et al.* [8]. This opened new fields of research in material science, magnetism and technology. Novel amorphous materials possessing unique combinations of properties (magnetic, mechanical, corrosion, *etc.*) such as metastable crystalline phases and structures, extended solid solubilities of solutes with improved mechanical and physical properties, nanocrystalline, nanocomposite and amorphous materials have been introduced [7,8]. Technological development of the fabrication techniques, structural characterization, studies of thermodynamics and physical properties (especially magnetic) of amorphous alloys were intensively performed in the 1960s and 1970s [9-11].

Most scientific, commercial and technological interest has been paid to magnetically soft amorphous and later — to nanocrystalline magnetic materials. Enhanced magnetic softness has been related to the absence of magnetocrystalline anisotropy in these amorphous alloys [11]. Particularly, combination of excellent soft magnetic properties of amorphous ribbons obtained by the melt-spinning technique with high wear and corrosion resistance made them very attractive for development of novel soft magnetic materials and for development of the applications in magnetic sensors, magnetic recording heads and the microtransformer industries [12].

Usually amorphous magnetic alloys exhibit extremely soft magnetic behaviour because of the absence of magnetocrystalline anisotropy, grain boundaries, and crystalline structure defects. Although crystallization usually results in degradation of magnetic softness of amorphous alloys, in some cases crystallization can improve magnetically soft behavior. This is the case of so-called “nanocrystalline” alloys obtained by suitable annealing of amorphous metals. These materials were introduced in 1988 by Yoshizawa *et al.* [13] and have been intensively studied later by a number of research groups [14,15]. Research and technological interest in such nanocrystalline alloys, also denominated “Finemet” (in the case of Fe-rich nanocrystalline alloys) arose from their extremely soft magnetic properties combined with high saturation magnetization. This nanocrystalline structure of partially crystalline amorphous precursors is observed in Fe-Si-B with small additions of Cu and Nb. It is widely assumed that the role of these small additions of Cu and Nb is to inhibit grain nucleation and decrease the grain growth rate [14,15]. The soft magnetic character is thought to be originated because the magnetocrystalline anisotropy vanishes and there is a very small magnetostriction value when the grain size approaches 10 nm [14,15]. As was theoretically estimated by Herzer [16], average anisotropy for randomly oriented α-Fe(Si) grains is negligibly small when grain diameter does not exceed about 10 nm. Thus, the resulting magnetic behavior can be well described with the random anisotropy model [16]. According to this model, low coercivity in the nanocrystalline state is ascribed to small effective magnetic anisotropy (K_eff_ around 10 J/m^3^). Low values of the saturation magnetostriction are essential to avoid magnetoelastic anisotropies arising from internal or external mechanical stresses. The increase of initial permeability with the formation of the nanocrystalline state is closely related to a simultaneous decrease of the saturation magnetostriction [17].

It is remarkable that a number of researchers have investigated the effect of the substitution of Fe in the Fe_73.5_Cu_1_Nb_3_Si_13.5_B_9_ alloy composition (the so-called Finemet*)* by an additional alloying element, like Co or Ni, in order to improve the magnetic properties [18]. Quite soft magnetic behaviour and GMI effect were observed in Finemet nanocrysalline ribbons where Fe has been partially substituted by Co [18].

Starting from the 90s a novel family of amorphous magnetic materials—amorphous wires—have been introduced [19,20]. The first generation of amorphous wire deals with typical diameters around 125 μm, obtained by the so-called in-rotating-water quenching technique. These materials exhibit a number of unusual magnetic properties. Thus, the positive magnetostriction compositions exhibit rectangular hysteresis loops, while the best magnetic softness is observed for the nearly-zero magnetostriction composition. Their main technological interest is related to the magnetic softness in nearly-zero magnetostriction composition, magnetic bistability in non-zero magnetostriction compositions and GMI effect [19-22]. This GMI effect consists of the large change of the electric impedance of a magnetic conductor when it is subjected to an axial DC magnetic field. It has been recognized that the large sensitivity of the total impedance of a soft magnetic conductor at low magnetic fields and high frequencies of the driven AC current originates from the dependence of the transverse magnetic permeability upon the DC magnetic field and the skin effect.

Generally, the GMI effect was interpreted in terms of the classical skin effect in a magnetic conductor assuming scalar character for the magnetic permeability, as a consequence of the change in the penetration depth of the AC current caused by the DC applied magnetic field. The electrical impedance, *Z*, of a magnetic conductor in this case is given by [23,24]:

(1) $$Z=R_{dc}krJ_{0}(kr)/2J_{1}(kr)$$

with *k = (1 + j)/δ,* where *J_0_* and *J_1_* are the Bessel functions, *r* –wire's radius and *δ* the penetration depth given by:

(2) $$\delta =\sqrt{\pi \sigma \mu_{\phi }f}$$

where *σ* is the electrical conductivity, *f* the frequency of the current along the sample, and *μ_ϕ_* the circular magnetic permeability assumed to be scalar. The DC applied magnetic field introduces significant changes in the circular permeability, *μ_ϕ_*. Therefore, the penetration depth also changes through and finally results in a change of *Z* [23,24]. Recently this “scalar” model was significantly modified taking into account the tensor origin of the magnetic permeability and magnetoimpedance [25].

It is worth mentioning that the shape of the magnetic field dependence of the GMI ratio, *ΔZ/Z,* at least for the small negative magnetostriction constant, has a non-monotonic shape with the maximum at the field corresponding to the circular magnetic anisotropy field. Therefore we assume that the shape of the magnetic field dependence of *ΔZ/Z* should be sensitive to the magnetic anisotropy especially in the surface layer of the sample. Besides, the magnetoelectric effects (inverse Wiedemann and Matteucci effects) have been widely used too [20,26].

**Figure 1.**
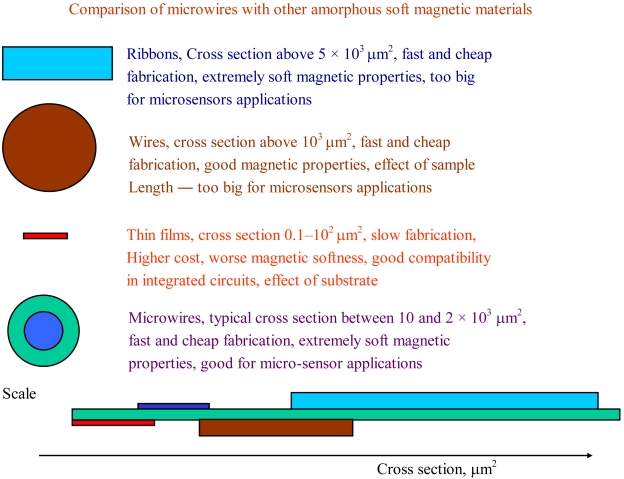
Scheme illustrating comparison of microwires with other soft magnetic materials [34].

**Figure 2.**
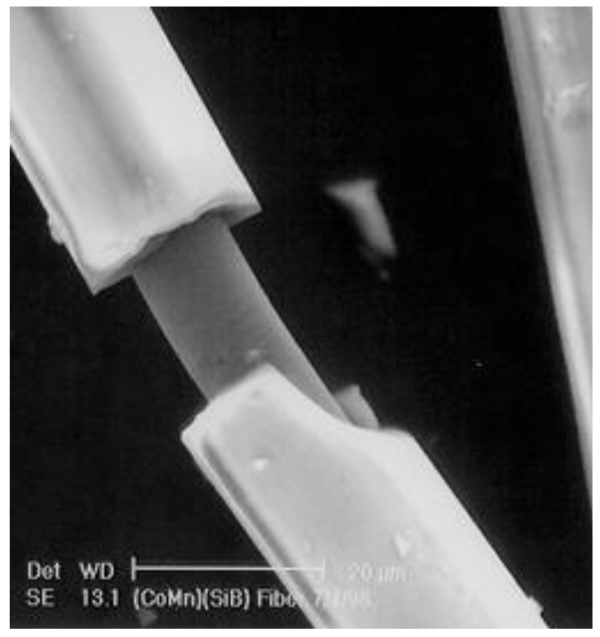
Micrograph of the glass-coated microwire [2].

The last advances in magnetic materials are based on the miniaturization of modern magnetic materials (see a comparison in Figure 1). The alternative technology of rapid quenching—the Taylor Ulitovski method—to produce thinner metallic wires (on the order of 1 to 30 μm in diameter) covered by an insulating glass coating has been widely employed for fabrication of ferromagnetic microwires coated by glass (see photo in Figure 2) since the mid-1990s [2-5,17,22]. It should be mentioned that the technology itself for the fabrication of metallic glass-coated microwires has been known since the 1950s and first reports on fabrication and properties of glass-coated microwires have been published in the 1970s. In fact, fabrication of short pieces of glass-coated microwires was reported by Taylor in 1924 (see more details in reference [2]), although significant modification of the technology through introduction of receiving spools allowing fabrication of km long glass-coated microwires was performed in the 1950s by Ulitovsky. Later, a cooling water jet has been introduced allowing rapid quenching of metallic alloy in order to produce amorphous glass-coating microwires [2].

The great advantage of these microwires is that the obtained diameter could be significantly lower than in the case of amorphous wires produced by the in-rotating-water method. But their magnetic properties are also quite different from those of “thicker” amorphous wires. Thus, although like in the case of “thicker wires” it was observed that Fe-rich compositions with positive magnetostriction constant show generally rectangular hysteresis loops, Co-rich negative magnetostrictive compositions have almost non-hysteretic magnetization curves and glass coating removal results in appearance of magnetic bistability [2-5]. This is because the glass coating introduces additional internal stresses due to the difference between the thermal expansion coefficients of glass coating and metallic nucleus. Therefore, microwires of the same composition can show different magnetic properties because of the different magnetoelastic energy. Depending on the thickness of the glass coating, the switching field (applied magnetic field necessary to observe magnetic bistability) is generally one order of magnitude higher than for melt-spun wires.

On the other hand, when the magnetostriction constant, *λ_s_*, is close to zero, a great variety of magnetic effects can be observed, depending on the sign of *λ_s_*. It has been found that the hysteresis loop changes from unhysteretic for slightly negative magnetostriction constant to rectangular for positive magnetostriction [2-5]. In the case of low positive magnetostriction constant some of experimental results were explained assuming the change of the sign of the magnetostriction constant under the effect of internal stresses [2].

Consequently, as a result these materials are very interesting for field and stress-sensing elements because the Fe-rich amorphous alloys exhibiting high magnetostriction values (*λ_s_* = 10^−5^) and therefore many of magnetic parameters (*i.e.*: magnetic susceptibility, coercive field…) are extremely sensitive to the applied stresses.

The other source of magnetic anisotropy can be the shape anisotropy. In the case of amorphous ribbons and wires this shape anisotropy can be significant. Thus, for Fe-rich amorphous wires with diameters of about 120 μm significant effect of the samples length on hysteresis loop has been observed for samples of 7 cm length [2]. It means that for sample lengths (denominated as critical length, *L_cr_*) below 7 cm, magnetically bistable behavior cannot be observed. In this sense, thin wires produced by the Taylor-Ulitovsky technique have a clear advantage: demagnetizing factors do not affect their magnetic behaviour if the sample length exceeds a few mm [2]. A critical length, *L_cr_*, to observe magnetically bistable was connected with the existence of “closure” domains at the ends of wires and was related with the penetration of the closure domains, *L_cd_*, inside the core as *L*_cr_ ≈ *2L_cd_*. Consequently, for a given sample (fixed diameter) the modification (*i.e.* decrease) of its length would result in a change (increase) of the demagnetizing factor. In other words, a decrease of diameter for a given length should result in a decrease of the closure domain penetration and of the critical length for bistability [2]. Particularly for the glass-coated microwire with metallic nucleus diameter, *d* ≈ 10 μm magnetically bistable behaviour has been observed for the sample length of 2 mm, *i.e.* for *d* ≈ 10 μm *L*_cr_ is below 2 mm. This means that for the sample length of glass-coated microwire with *d* ≈ 10 μm, *L* > 2 mm, the ample length and consequently the demagnetizing factors do not affect overall magnetic behavior (hysteresis loop shape).

Circular domain structure with high circumferential permeability proved to be very favourable for the highest GMI effect [2-4,6]. Such a domain configuration is typical for the nearly-zero magnetostrictive amorphous wires mainly produced by Unitika Ltd [19,20]. In addition, it was recently demonstrated that the applied tensile stresses can significantly modify the magnetoimpedance response of conventional amorphous wires and glass coated microwires, especially if previously stress induced magnetic anisotropy has been induced [27-29]. On the other hand magnetic domain wall propagation becomes a hot topic of research because of its use in magnetic devices (such as Magnetic Random Access Memory, Integrated Circuits, Hard Disks, *etc.*) to transmit the information along the magnetic wire of submicrometer diameter [30,31].

In the case of magnetic microwires with positive magnetostriction constant the magnetic bistability characterized by the appearance of rectangular hysteresis loops at low applied magnetic field has been observed [5,32,33]. This magnetic bistable behavior is related to the presence of a single Large Barkhausen Jump, which was interpreted as the magnetization reversal in a large single domain [2-6,32,33]. Such a peculiar remagnetization process is quite interesting for various applications [2,34]. It is important, that a single and large Barkhausen jump is observed above some critical fields regarding the critical length, and it can be correlated well with the demagnetizing factor [33] indicating that the closure domains penetrate from the wire ends inside the internal axially magnetized core destroying the single domain structure. For the case of commercially available amorphous wires (with diameter about 120 μm) this critical length is about 7 cm, which is quite inconvenient for use in magnetic micro-sensors and microelectronics. In glass-coated microwires with diameter about 10 μm this critical length is much shorter (about 2 mm) which is quite suitable for microsensor applications. This rectangular hysteresis loop also disappears when the magnetic field is below some critical value denominated as the switching field [33]. Such a rectangular hysteresis loop was interpreted in terms of nucleation or depinning of the reversed domains inside the internal single domain and the consequent domain wall propagation [5,32-34]. Perfectly rectangular shape of the hysteresis loop has been related with a very high velocity of such domain wall propagation. It was demonstrated that the remagnetization process of such magnetic microwire starts from the sample ends as a consequence of the depinning of the domain walls and subsequent DW propagation from the closure domains [5,32-34]. Quite surprising results such as the existence of the domain wall propagation below the switching field [32], negative critical propagation field [5] and supersonic domain wall propagation [5] have been reported.

Another field of applied research is related with development of composite materials with short pieces of embedded thin ferromagnetic wires have been recently introduced [35,36]. The electromagnetic properties of such metamaterials can be very sensitive to external DC magnetic fields, applied stress or temperature in the microwave range. The wire inclusions play a role of “the elementary scatterers”, when the electromagnetic wave irradiates the composite and induces a longitudinal current distribution and electrical dipole moment in each inclusion. These induced dipole moments form the dipole response, which can be characterized by some complex effective permittivity. The latter may have a resonance or relaxation dispersion caused by the strong current distribution along a wire, which depends on the wire high frequency surface impedance. In the vicinity of the resonance frequency any variations in the wire surface impedance result in a large change of the current distribution, and hence in the dipole moment of each inclusion and the effective permittivity on the whole. For a ferromagnetic conductive wire, the surface impedance may depend not only on its conductivity but also on the DC external magnetic field and tension through the so-called magneto-impedance (MI) effect. Therefore, the dispersion of the effective permittivity can be tuned when a sufficient magnetic field or tensile stress is applied to the composite sample. In this paper we present overview of magnetic and magneto-transport properties of thin wires relevant for technological applications

## 2. Results and Discussion

### 2.1. Effect of composition. Properties relevant for applications

**Figure 3.**
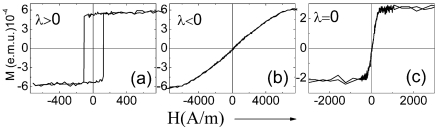
Hysteresis loops of Fe-rich (*λ_s_* > 0), Co-rich (*λ_s_* < 0) and Co-Fe-rich *(λ_s_* = 0) microwires [38].

The magnetic properties and overall shape of hysteresis loops of amorphous microwires depend on composition of the metallic nucleus as well as on thickness of the glass coating. It should be mentioned that even the composition of the glass coating affects the magnetic properties of glass-coated microwires [37]. The effect of metallic nucleus composition on magnetic properties and hysteresis loop shape is illustrated by Figure 3, where the hysteresis loops of three main groups of amorphous microwires (Fe-rich, Co-rich and Co-Fe-rich with positive, negative and vanishing magnetostriction constant, *λ_s_*, respectively) are shown.

The shape of the hysteresis loops changes from the rectangular one typical of amorphous Fe-rich compositions to the inclined one typical of Co-rich compositions. Microwires with vanishing *λ_s_* exhibit quite soft magnetic properties.

**Figure 4.**
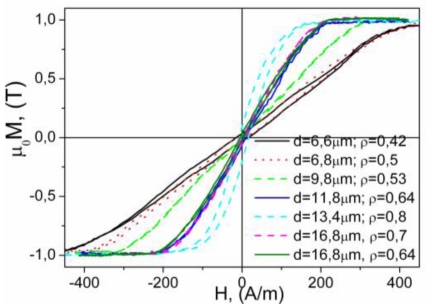
Hysteresis loops of Co_67_Fe_3.85_Ni_1.45_B_11.5_Si_14.5_Mo_1.7_ microwires with different ρ–ratio [34].

If the composition of the metallic nucleus is the same, the thickness of glass coating also affects magnetic properties. Thus, Figure 4 shows hysteresis loops of amorphous microwires with the same composition (Co_67_Fe_3.85_Ni_1.45_B_11.5_Si_14.5_Mo_1.7_ with vanishing *λ_s_*) but with different ρ-ratio defined as *d/D* (where *d* = metallic nucleus diameter, *D* = total microwire diameter). In the case of nearly-zero magnetostrictive Co_67_Fe_3.85_Ni_1.45_B_11.5_Si_14.5_Mo_1.7_ microwires, all samples exhibited inclined *M(H)* loops with extremely low coercivities (up to 4 A/m) but small magnetic anisotropy fields, *H_k_*. The magnetic anisotropy field, *H_k_*, increases with increasing the ρ-ratio (Figure 4).

Such a strong dependence of the hysteresis loops and magnetic anisotropy field on these parameters should be attributed to the magnetoelastic energy given by:

(3) $$K_{me}\approx \frac{3}{2}\lambda_{s}\sigma_{i},$$

where *σ_i_* is the internal stress. The magnetostriction constant depends mostly on the chemical composition and vanishes in amorphous Fe-Co based alloys with Co/Fe ≈ 70/5 [2]. On the other hand, the estimated values of the internal stresses in these glass coated microwires arising from the difference in the thermal expansion coefficients of metallic nucleus and glass coating are of the order of 100–1,000 MPa, increasing with the increase of the glass coating thickness [2-4,6]. Such large internal stresses give rise to a drastic change of the magnetoelastic energy, *K_me_*, even for small changes of the glass-coating thickness at fixed metallic core diameter. Additionally, such a change of the ρ-ratio should be related to the change of the magnetostriction constant with applied stress [2,4,34]:

(4) $$\lambda_{s}=\frac{\mu_{0}M_{s}}{3}\frac{dH_{k}}{d\sigma },$$

where *μ_0_M_s_* is the saturation magnetization, *H_k_*- magnetic anisotropy field.

The main interest has been directed towards studies of Fe-rich compositions with positive λ_s_ exhibiting perfectly rectangular hysteresis loop (so-called magnetic bistability) and Co-rich compositions with nearly-zero *λ_s_* with good magnetic softness. This is because soft magnetic microwires, exhibiting high magnetic permeability and also GMI effect are quite useful for applications in magnetic field sensors [2-5,34]. On the other hand microwires with rectangular hysteresis loops can be used for magnetic sensors for magnetic surveillance and for magnetic tags [2,5,34].

### 2.2. Magnetic bistability

One of the main technological interest for utilization of amorphous microwires is related with the Large and single Barkhausen Jump (LBJ) [2,33,34,38]. It is worth mentioning that appearance of LBJ takes place under magnetic field above some critical value (called the switching field) and also if the sample length is above some critical value also known as the critical length. The switching field depends on the magnetoelastic energy *K_me_* [2-4,33,34]. Regarding the critical length, detailed studies of the ferromagnetic wire diameter on magnetization profile and size of the edge closure domains have been performed in [2,33,34,38]. Particularly, critical length, *L_cr_*, for magnetic bistability in conventional Fe-rich samples (120 μm in diameter) is about 7 cm. This critical length depends on saturation magnetization, magnetoelastic energy, domain structure, magnetostatic energy [2,33,34,38].

Enhanced critical length, *L_cr_*, (of the order of few cm) observed in conventional amorphous wires has limited sensor applications. Reduction of the metallic nucleus diameter in the case of glass-coated microwires (almost one order lower) results in drastic reduction of the critical length, making them quite attractive for micro-sensor applications. Thus, magnetic bistability for the sample length *L* = 2 mm has been observed for Fe-rich microwire with metallic nucleus diameter, *d*, about 10 μm [33].

### 2.3. Fast domain wall propagation

Recently great attention has been paid to studies of domain wall (DW) propagation in thin wires with sub-micrometric and micrometric diameter [30,31]. Growing interest in DW propagation is related to proposals for prospective logic and memory devices [39-41]. The speed at which a DW can travel in a wire has an impact on the viability of many proposed technological applications in sensing, storage, and logic operation. Special effort has been performed on nanowires to enhance the DW speed. Thus, application of transverse magnetic field proposed in [42] allowed one to slightly improve the DW speed, *v*, to about 600 m/s.

**Figure 6.**
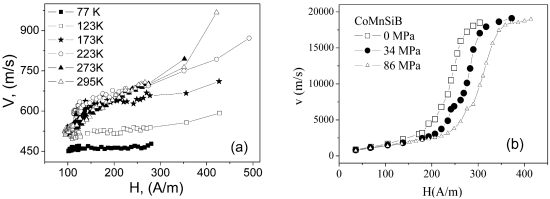
(a): *v(H)* dependence measured for the Fe_69_Si_10_B_15_C_6_ microwire with the diameter of metallic nucleus, *d* = 14 μm at different temperatures, *T* and (b): for the Co_68_Mn_7_Si_10_B_15_ microwire with *d* = 8 μm at different applied stress [5,43].

Similarly, we observed DW propagation in ferromagnetic microwires with rectangular hysteresis loops [5,32,43]. We studied the DW dynamics by classical Sixtus-Tonks experiments [44], as described recently elsewhere [5,32,43]. The dependence of *v* on applied magnetic field, *H*, measured for the Fe_69_Si_10_B_15_C_6_ microwire with the diameter of metallic nucleus 14 μm at different temperatures, *T*, is shown in Figure 6a.

Quite high DW velocity, *v*, and essentially non-linear magnetic field dependence, *v(H)* is observed. Even higher DW propagation has been observed in Co_68_Mn_7_Si_10_B_15_ (with the diameter of the metallic core being 8 μm and total diameter being 20 μm) which is characterized by the lowest possible magnetoelastic anisotropy (due to its low magnetostriction *λ_s_*) among the microwires with single domain axial structure (see Figure 6b).

Observed non-linearity of *v(H)* dependences at low field related with change of the regime from the lower field with smaller DW velocity to the higher field with enhanced velocity at some critical field, *H_cr_*, can be attributed to the change of the structure of the DW as well as to the adiabatic domain wall propagation at magnetic field slightly above critical field, attributed to the interaction of the DW with the local defects [45].

Regarding the change of the DW domain wall velocity due to the change of the DW structure, it was shown previously by micromagnetic simulations for the case of nanowires that the vortex-type domain wall is faster than the transversal one and the vortex domain wall is more stable for the case of thicker magnetic wires [5].

**Figure 7.**
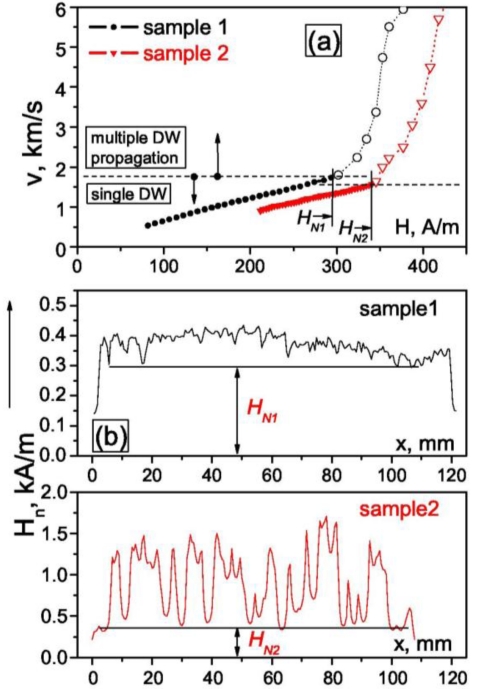
(a) *v(H)* Dependences measured in magnetically bistable Fe_75_Si_12_B_9_C_4_ microwires and (b) distribution of local nucleation fields *H_n_* measured in the same samples.

On the other hand, an abrupt increase of DW velocity is observed at high field region (see Figure 7) and can be explained by nucleation of additional DWs on local defects existing inside the microwire [46,47]. Such local defects have been found using short magnetizing coils placed far from the wire ends [46]. In this case, nucleation of new DW far from the wire end can be realized. Consequently much higher magnetic field is needed for such nucleation as-compared with the depining of the DW from the wire ends (Figure 7b). Moving the magnetizing coil along the wire we observed spontaneous fluctuations of local nucleation field *H_n_* which is very sensitive to the presence of the local defects. The overall minimum *H_N_ = min(H_n1_, H_n2_,…)* correlated very well with the field of abrupt increasing of *v* (Figure 7a). Consequently, the defects existing in microwires play a role of the nucleation centers for new DWs. When the applied magnetic field exceeds *H_N_*, new reverse domains can be nucleated, which results in s significant decrease of the magnetization switching time and acceleration of magnetization switching in magnetically bistable microwires. This mechanism of ultrafast magnetization switching through additional nucleation centers created artificially can be applied in spintronic devices for enhancing their performance.

### 2.4. Giant magneto-impedance effect and enhanced magnetic softness. Tailoring of magnetic properties and GMI

**Figure 8.**
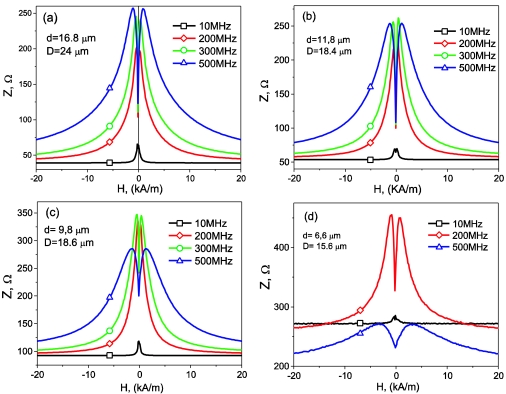
*Z(H)* dependence Co_67_Fe_3.85_Ni_1.45_B_11.5_Si_14.5_Mo_1.7_ microwires with different geometry [34].

Usually the GMI effect is characterized by the magnetoimpedance ratio, *ΔZ/Z*, has been defined as:

(5) $$\frac{\Delta Z}{Z}=\frac{Z(H)-Z(H_{\max})}{Z(H_{\max})}$$

where *H_max_* is the maximum DC longitudinal magnetic field of the order of few kA/m, usually supplied by a long solenoid and/or Helmholtz coils. The appearance of the GMI effect and magnetic field dependence of impedance are intrinsically related with the magnetostriction constant, domain structure and above-mentioned outstanding magnetic softness of magnetic materials. As has been observed, hysteresis loops are quite sensible to the chemical composition and sample geometry (see Figures 3 and 4). Until now, the best soft magnetic properties and highest GMI effect have been observed in Co_67_Fe_3.85_Ni_1.45_B_11.5_Si_14.5_Mo_1.7_microwires [3]. Hysteresis loops presented in Figure 3c exhibit inclined almost unhysteretic *M(H)* loops with extremely low coercivities (up to 4 A/m). Magnetic anisotropy field, *H_k_*, increases with increasing ρ-ratio (Figure 4). As expected we observe (see Figure 8) that the sample geometry strongly affects the DC axial magnetic field dependence of the real part of GMI, *Z* of Co_67_Fe_3.85_Ni_1.45_B_11.5_Si_14.5_Mo_1.7_ microwires.

Since it is known that the strength of the internal stresses is determined by the ρ-ratio, the relaxation of such internal stresses by means of thermal treatment should drastically change both soft magnetic behaviour and *ΔZ/Z(H)* dependence.

**Figure 9.**
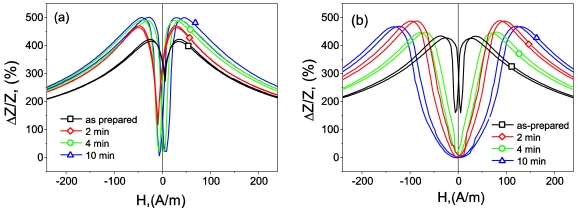
*ΔZ/Z(H)* dependences measured at (a) *f* = 30 MHz and *I* = 1 mA in annealed at 30 mA and (b) at 40 mA of Co_67_Fe_3.85_Ni_1.45_B_11.5_Si_14.5_Mo_1.7_ microwire [34].

Figure 9 shows this dependence measured for as-prepared and annealed Co_67_Fe_3.85_Ni_1.45_B_11.5_ Si_14.5_Mo_1.7_ samples. Both *ΔZ/Z* and the DC-field, *H_m_*, corresponding to the maximum of the GMI ratio, can be modified by annealing.

The development of miniaturized sensors requires microwires with thinner diameter but keeping their good soft magnetic properties and GMI effect. Consequently, enhanced soft magnetic properties (coercivity below 10 A/m) and considerable GMI effect observed in thin microwires with metallic nucleus diameter, *d*, below 10 μm should be considered as a significant achievement in development of materials for micro-sensor applications.

**Figure 10.**
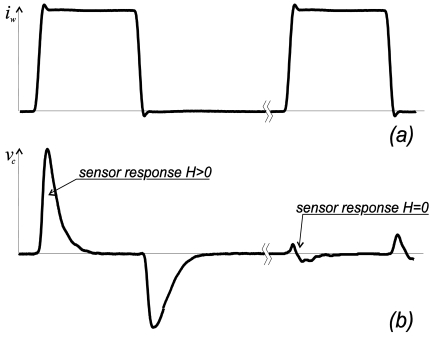
Excitation current pulse in the wire (a) and voltage induced in the pickup coil (b).

Recently it was found that off-diagonal compo­nents possess asymmetrical dependence on the magnetic field, the necessary condition for determination the magnetic field direction [3]. For practical sensors the pulsed excitation is preferred over a sinusoidal one because of the simple elec­tronic design and low power consumption. The practical circuit design [48] consists of a pulse generator, sensor element and output stage. The sharp pulses are produced by passing squared wave pulses through the differential circuit. When the pulse flows through the magnetic wire the magnetic field dependent signal, *V_out_*, appears in the pickup coil as shown in Figure 10.

**Figure 11.**
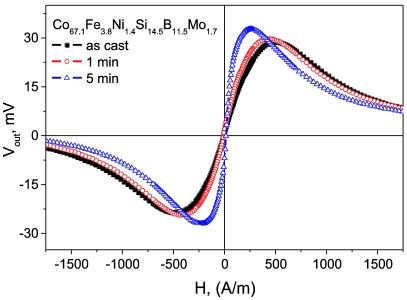
*V_out_(H)* of Joule-heated Co_67_Fe_3.85_Ni_1.45_B_11.5_Si_14.5_Mo_1.7_ microwire annealed with 50 mA currents for different time [3].

Figure 11 shows field dependence of the off-diagonal voltage response, *V_out_*, measured as described elsewhere [3,34,48] in Co_67_Fe_3.85_Ni_1.45_B_11.5_Si_14.5_Mo_1.7_ (*λs* ≈ 3 × 10^−7^) microwire. The *V_out_(H)* curves have asymmetrical shape exhibiting close to linear growth within the field range from *−H_m_* to *H_m_* (Figure 11). The *H_m_* limits the working range of MI sensor to 240 A/m and should be associated with the anisotropy field.

The influence of Joule heating on off-diagonal field characteristic of nearly zero magne­tostriction Co_67_Fe_3.85_Ni_1.45_B_11.5_Si_14.5_Mo_1.7_ microwire with diameters 9.4/17.0 μm is shown in Figure 11. One can see that the thermal annealing with 50 mA DC current reduces the *H_m_* from 480 A/m in as-cast state to 240 A/m after 5 min annealing.

**Figure 12.**
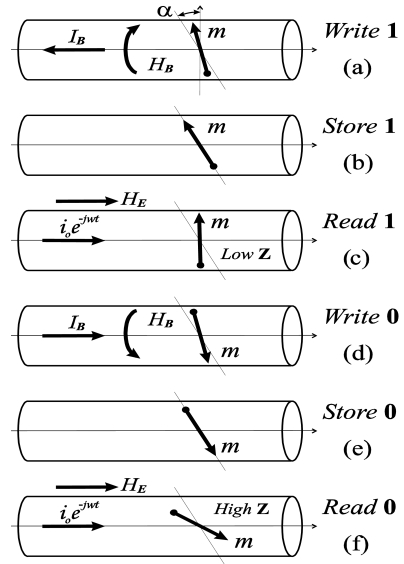
Principle of data storage in wire element. α is the angle between the anisotropy easy axis and the transversal plane.

One of the main features that can affect the magnetic field sensor's resolution is related with the MI hysteresis, as can be seen in Figures 9 and 12. The origin of this hysteresis is related to the deviation of the anisotropy easy axis from the circular direction in the wire's outer shell. To suppress the hysteresis a circular bias field *H_B_* produced by the DC current can be used. Although this effect can be turn out to be very interesting for certain applications. For example, the hysteresis can be used for data storing.

The storage and erasing of the information can be performed simply by passing the current pulse through the wire while the stored data is retrieved by measurement of the wire's impedance in the presence of the constant magnetic field. The principle is shown in Figure 12. Independently of the initial state, after applying the constant current pulse *I_B_* in one direction (from right to left in Figure 12a), the spins will orient along the easy axis in *up* direction (Figure 12b) which corresponds to the store logical ‘1’ state. Then, under applied external magnetic field makes spins rotate towards *close to circumferential* direction which characterized by low impedance (Figure 12c) that can be easily detected. To write logical '0' one need to pass current pulse *I_B_* in the opposite direction (left to right in Figure 12d) that makes the spins aligned along the easy axis in *down* direction (Figure 12e). In this case, under application of the external magnetic field the spins rotate towards *close to longitudinal* direction characterized by high impedance as shown in Figure 12f. The change of impedance between the *Low-Z* and *High-Z* states about 50% or even higher might be observed. The main advantage of the proposed method for data storage is the absence of moving components such as the recording and writing heads that found in hard disk drives.

### 2.5. Applications of thin magnetically soft wires. Sensor prototypes

The phenomenon of magnetic bistability is characterized by the sharp voltage peaks induced in the pick-up coil wound around the sample. The induced *emf* is caused by an abrupt change of the magnetic flux during the large Barkhausen jump. This effect can be used in magnetic sensors. The first sensors based on magnetic bistability (MB) were introduced in the 1970s, when Wiegand wires [49] with rectangular hysteresis loops inducing sharp 20–30 μs pulses in the secondary coil mounted on the sample subjected to the AC magnetic field were developed. Such sensors were widely applicable in the automobile industry for the detection of the motions and positions [2,34]. However, such Wiegand wires need relatively large excitation fields (about 4 kA/m) to produce sharp voltage peaks and special processing. Therefore, amorphous Fe-rich (Fe_80_B_20_ or Fe_81_B_17_Si_2_) magnetostrictive ribbons subjected by the special stress annealing were introduced at the beginning of the 1980s [50]. Stress annealing was performed in the samples of toroidal shape in order to induce tensile-stressed and compressive-stressed layers in the ribbon. Sharp voltage pulses with 20–50 μs duration at field amplitude of around 100 A/m at frequency of 0.1–6 Hz were found in these amorphous ribbons. Such magnetic bistability was initially used for the magnetometer and rotation speed sensors [50]. Later, the Matteucci effect, appearing in the twisted amorphous ribbon was also employed for the modified rotation speed sensor [51]. Like in the case of sensors based on magnetic bistability, sharp voltage pulses appears between the sample ends when it is submitted to magnetic field that was used for sensor design [51]. In this case the secondary coil was not necessary.

Throughout the 80s and later, an increasing interest has been focused on amorphous materials with a cylindrical shape. The in-rotating-water fabrication technique was widely employed for the production of around 120 μm amorphous wires. It was found that either as-cast or die-drawn then annealed magnetostrictive amorphous wires exhibit the re-entrant flux reversal characterized by large and single Barkhausen jump [2,34]. Consequently, a number of magnetic sensors based on magnetic bistability and/or Matteucci effect of amorphous wires were developed [2,19-21,34]. As it was expected, sharp voltage peaks appearing in the secondary coil or between the sample ends are commonly used for the distance sensor, revolution counter and position sensor [2,19-21]. On the other hand, such peculiar remagnetization process with large Barkhausen discontinuity exhibiting by amorphous wires has been used for design of magnetic markers and tags [52,53]. In these applications a consequence of voltage peaks appearing under the application of the external magnetic field is used. General problem of such applications is the limited number of combination for the good identification due to the similarity of the switching field values. In order to extend the switching field range, a coating by the hard magnetic materials is recently used [54].

Later it was found that thinner glass-coated amorphous microwires can also exhibit magnetic bistability effect even for samples with a length of a few millimeters and for nearly zero-magnetostrictive alloys [2-4,33,34], while the negative magnetostriction compositions do not exhibit magnetic bistability. It is important to outline that even Fe-rich glass coated microwires exhibit a wide range of switching fields. Besides such wide range of the switching field can be extended by the thermal treatments [2,17,34,38,55].

**Figure 13.**
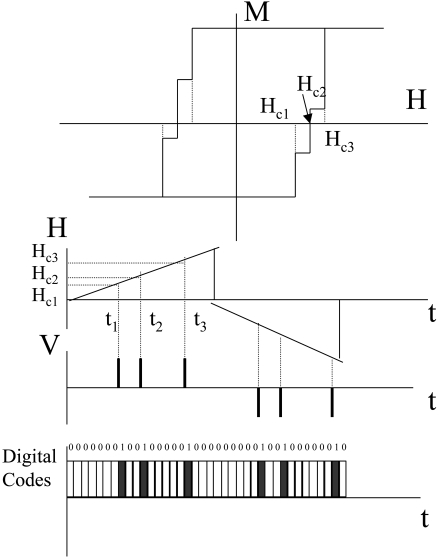
Schematic representation of the encoding system based on magnetic bistability of the microwires [38].

Magnetic bistability with extended switching field range and high stress sensitivity of magnetic parameters (switching, field) give rise to various technological applications of tiny magnetic wires coated by glass [2,34,38,55]. One of the applications is based on the wide range of coercivity, which can be obtained in microwires owing to its strong dependence on geometric dimensions and heat treatment. It was realized in the method of magnetic codification using magnetic tags [2,34,38,55]. The tag contains several microwires with well-defined coercivities, all of them characterized by a rectangular hysteresis loop. Once the magnetic tag submitted to the AC magnetic field, each particular microwire is remagnetized at different magnetic field giving rise to an electrical signal on a detecting system (see Figure 13). The extended range of switching fields obtained in Fe-rich microwires gives a possibility to use a big number of combinations for magnetic codification [2,34,38].

**Figure 14.**
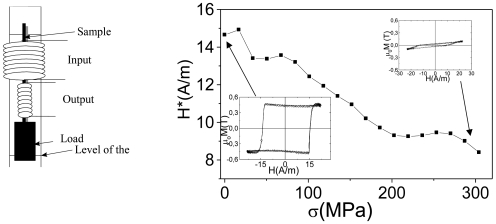
Schematic representation of the magnetoelastic sensor based on stress dependence of the switching field [55].

A magnetoelastic sensor of the level of a liquid can be designed using the stress dependence of the coercivity in nearly-zero magnetostriction CoMnSiB microwires. Such a sensor essentially consists of a piece of the wire surrounded by primary and secondary coils (Figure 14) [2,34,38,56]. The sample is loaded with approximately 10 grams and therefore exhibits a flat hysteresis loop. The weight is attached to the bottom of the sample. When a liquid arrives to cover the weight as a consequence of the actual stress, affecting the sample, decreases giving rise to the appearance of the rectangular hysteresis loop.

The principle of the sensor's work is based on the change of the voltage of the secondary coil, which increases drastically when the hysteresis loop of the wire became rectangular after the stress essentially decreases. A simple circuit including the amplification of the signal and alarm set was used to detect changes of voltage in the secondary coil under the change of tensile stresses owing the floating effect of the weight on the liquid.

**Figure 15.**
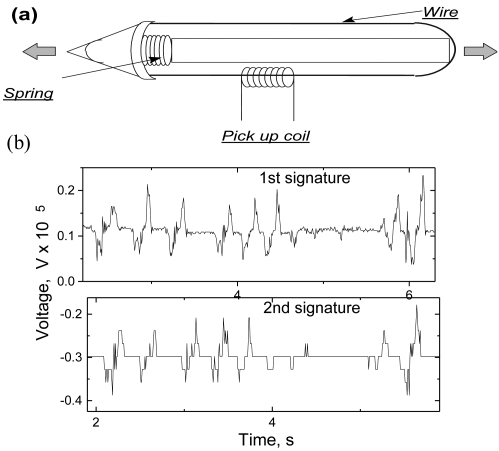
Schematic representation of the magnetoelastic pen (a) and two magnetoelastic signatures (b) [55].

The observed magnetic response on the external variable stresses can be used in many applications to detect different temporal changes of stresses, vibrations *etc*. As an example we introduce a “magnetoelastic pen” which can be used for identification of signatures [2,34,38,55,57]. It is known that the signature of each person can be represented by a typical series of stresses. The sequence and strength of those stresses are a characteristic feature of any personal signature, so temporal changes of the stresses while signing can be used for the identification of signature itself. We have used this behavior and designed a set-up consisting of a ferromagnetic bistable amorphous wire with positive magnetostriction, a miniaturized secondary coil and a simple mechanical system inside the pen containing a spring, which transfers the applied stresses to the ferromagnetic wire (see Figure 15). The resulting temporal dependence of the stresses, corresponding to a signature is reproducible for each individual person. The main characteristics of this dependence are time of signature, sign and sequence of the detected peaks. Examples of two magnetoelastic signatures corresponding to the same person are shown in the Figure 15.

Since the discovery of the magneto-impedance effect in 1994 [23,24], a number of studies and developments on this topic have been widely performed by different groups [2-4,34,55]. As a result, GMI and stress-impedance (SI) sensors with CMOC IC circuitry with advantageous features compared with conventional magnetic sensors have been developed [58,59]. Among the industrial applications, MI element using amorphous wire, electronics compass using MI element incorporated into mobile phones and accelerometers using MI sensor and 6 axis type of motion sensor which combine 3 axis magnetic sensors with 3 axis accelerometers have been reported by Aichi Steel Co. [58,59]. The sensor's circuitry, performance and comparative characteristics with the traditional sensors are presented in [58,59]. Similarly, the other sensors based on stress sensitivity of the GMI effect for the domestic use have been developed in Spanish groups [60].

As an example the air flux sensor is described below. This sensor is based on the stress sensitivity of GMI effect exhibited by magnetically soft Co_68.5_Mn_6.5_Si_10_B_15_ microwire. In the unstressed state GMI ratio, *ΔZ/Z*, of Co_68.5_Mn_6.5_Si_10_B_15_ microwire exhibits a maximum on magnetic field, *H*, dependence, *ΔZ/Z(H)* at around *H* = 120 A/m of axial applied field. This maximum displaces towards larger applied fields when external stress, σ, is applied.

**Figure 16.**
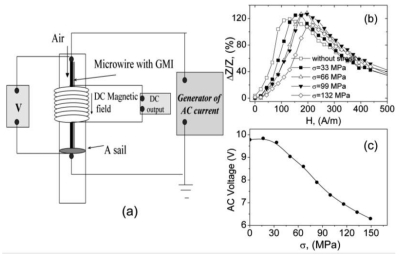
Schematic representation of the magnetoelastic sensor based on stress dependence of GMI effect (a), *ΔZ/Z(H)* dependencies of CoMnSiB amorphous microwire measured at different applied stress (b) and calibration curve of sensor (c) [55].

This high sensitivity of the GMI ratio to applied stress makes this stress sensitive GMI effect to be very interesting for practical purposes. Figure 16 shows a sensitive magnetoelastic device based on GMI exhibited by this microwire with the following working principle: the microwire has rather high DC electrical resistance per length owing to its tiny dimensions. Therefore, large changes of the magnetoimpedance could induce rather large changes of the AC voltage under mechanical loading. Indeed, under the effect of tensile stresses, the GMI ratio at fixed applied field, *H*, decreases giving rise to a significant change of the AC voltage between the sample's ends.

A DC magnetic field corresponding to the maximum ratio *(ΔZ/Z)_m_* without stresses has been applied by means of a small solenoid. The change of AC voltage (peak to peak) between the ends of the sample is of around 3.5 V for small mechanical loads attached at the bottom of the microwire (see calibration curve in the Figure 16c). This huge change presented at the output permits one to conceive the successful use of this kind of sensor in different technological applications related with the detection of alternative mechanical stress.

**Figure 17.**
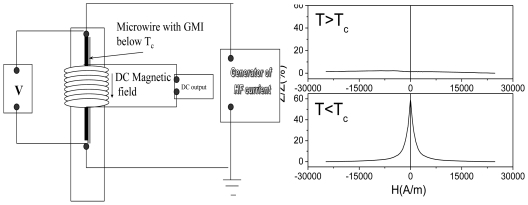
Schematic picture showing the working of the temperature sensor based on GMI effect in microwires with low *T_C_* [34].

**Figure 18.**
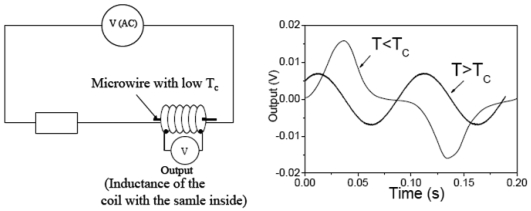
Schematic picture showing the working of the temperature sensor based on change of the inductance in microwires with low *T_C_* [34].

Among recent applications, temperature dependence of the GMI effect and magnetic susceptibility in microwires with low Curie temperature, *T_C_*, can be mentioned [34]. Particularly, the functioning of the temperature sensor based on GMI effect is based on the drastic drop of the GMI effect after achieving the Curie temperature (see Figure 17). In this case the GMI effect of microwire disappears above the Curie temperature, which is detected by the proposed circuitry. On the other hand the schematic picture showing the principle of the sensor based on the change of the inductance of the coil with the microwire inside is shown in Figure 18. In this case, the inductance of the coil with ferromagnetic wire inside drastically changes above Curie temperature of the microwire, allowing detection of the temperature.

**Figure 19.**
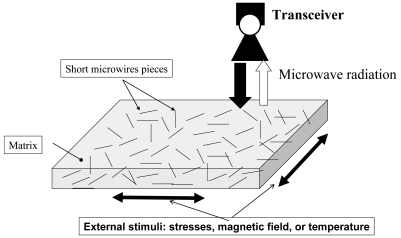
Free-space microwave sensing technique using embedded short ferromagnetic microwires [34].

On the other hand, as mentioned above, new types of magnetic field-tuneable, stress-tuneable and temperature-tuneable composite materials based on thin ferromagnetic wires with the effective microwave permittivity depending on an external DC magnetic field, applied stress or temperature recently have been introduced [34-36]. Such composites consist of arrays of continuous or short-cut (Figure 19) pieces of conductive ferromagnetic wires embedded into a dielectric matrix. Using magnetic microwires makes it possible to engineer low density materials with relatively high values of the effective magnetic permeability originated from natural magnetic properties of the wires with a circumferential magnetic anisotropy. The magnetic field in the incident wave along the wire will generate substantial magnetic activity as it will be in the orthogonal position with respect to a static magnetization.

The possibility to control or monitor the electromagnetic parameters (and therefore scattering and absorption) of the composites is of great interest for large-scale applications such as remote non-destructive testing, structural health monitoring, tunable coatings and absorbers. A number of applications have been proposed (as, for example, shown in Figure 19), including stress-sensitive media for remote non-destructive health monitoring of different structures, temperature-dependent media and selective microwave coatings with field-dependent reflection/transmission coefficients [34-36]. The important advantage of such applications is that the soldering problems are avoided because of the non-contact detection of the signals. It is worth mentioning that thin wires with stress sensitive magnetic anisotropy exhibiting stress sensitive GMI effect and SI effect are quite necessary for designing such composites.

On the other hand magnetic domain wall propagation has become a hot research topic because of the possibility of applications in magnetic devices, such as Magnetic Random Access Memory, Integrated Circuits, Hard Disks, Domain-wall Logics, *etc*. [30,31]. The high velocity of domain wall propagation observed in glass-coated microwires can be attractive to transmit the information along the magnetic microwire, like it was observed recently in wires of submicrometer diameter [30,31]. Clear advantage of glass-coated microwires is that the domain wall velocity is few times higher, achieving few km/s [5,34,43,45,47].

## 3. Conclusions

To summarize, the alloy composition and the additional magnetoelastic energy related with the presence of the glass coating require more attention for tailoring of magnetic properties of microwires. Post fabrication processing, *i.e.* special thermal treatments, precise control of their geometry, chemical composition of the metallic nucleus *etc*. From the point of view of magnetic properties, excellent magnetic softness can be achieved in these glass-coated microwires after rigorous control of their parameters (geometry, chemical composition *etc.*) and using post fabrication processing of such materials.

Quite fast domain wall propagation has been observed in glass-coated microwires. Essentially non-linear dependence of the DW speed on magnetic field has been observed. The origin of such dependences and high domain wall velocity is discussed in terms of magnetoelastic energy contribution (particularly of transverse magnetic anisotropy contribution) and interaction of domain walls with internal defects as well as adiabatic domain wall propagation.

Thin amorphous wires with excellent magnetic softness and large magnetoimpedance and stress impedance effects have been developed. It is demonstrated that the magnetic properties can be tailored by an appropriate selection of the alloy composition, wire geometry and thermal treatments. Stress annealing of Fe-rich glass-coated microwire makes it possible to considerably change the effective anisotropy and magnetic configuration. By varying the annealing time and temperature, an optimal magnetic configuration in Fe-rich microwires is achieved in terms of magnetic anisotropy, magnetic softness, GMI and SI. A number of interesting sensor prototypes using thin magnetically wires and few industrial sensor applications have been already developed.

## Figures and Tables

**Figure 5.**
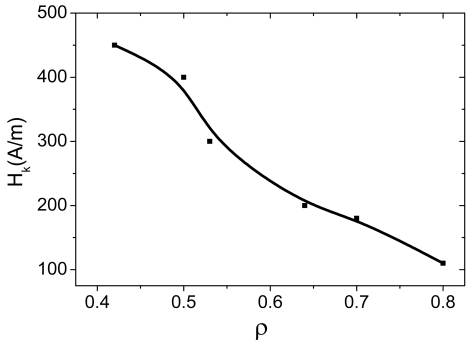
Effect of sample geometry (*ρ–*ratio) on magnetic anisotropy field, *H_k_* [34].
